# Supplementary material for: Preservation of lymphocyte functional fitness in perinatally-infected and treated HIV+ pediatric patients displaying sub-optimal viral control
Source: Commun Med (Lond). 2022 Mar 4;2:25. doi: 10.1038/s43856-022-00085-9 (PMC9012494; doi:10.1038/s43856-022-00085-9)
Supplement: Supplementary file 1 — Description of Additional Supplementary Files [file 43856_2022_85_MOESM1_ESM.pdf]

Description of Additional Supplementary Files

File Name: Supplementary Data

Description: Source Data for Figures 1-7
