# Supplementary material for: Preservation of lymphocyte functional fitness in perinatally-infected and treated HIV+ pediatric patients displaying sub-optimal viral control
Source: Commun Med (Lond). 2022 Mar 4;2:25. doi: 10.1038/s43856-022-00085-9 (PMC9012494; doi:10.1038/s43856-022-00085-9)
Supplement: Supplementary file 5 — Reporting Summary [file 43856_2022_85_MOESM5_ESM.pdf]

nature research

Corresponding author(s): Aaruni Khanolkar

Last updated by author(s): Jul 13, 2021

Reporting Summary

Nature Research wishes to improve the reproducibility of the work that we publish. This form provides structure for consistency and transparency in reporting. For further information on Nature Research policies, see our Editorial Policies and the Editorial Policy Checklist.

Statistics

For all statistical analyses, confirm that the following items are present in the figure legend, table legend, main text, or Methods section.

n/a	Confirmed	
☐	☒	The exact sample size (n) for each experimental group/condition, given as a discrete number and unit of measurement
☐	☒	A statement on whether measurements were taken from distinct samples or whether the same sample was measured repeatedly
☐	☒	The statistical test(s) used AND whether they are one- or two-sided<br>*Only common tests should be described solely by name; describe more complex techniques in the Methods section.*
☒	☐	A description of all covariates tested
☐	☒	A description of any assumptions or corrections, such as tests of normality and adjustment for multiple comparisons
☐	☒	A full description of the statistical parameters including central tendency (e.g. means) or other basic estimates (e.g. regression coefficient) AND variation (e.g. standard deviation) or associated estimates of uncertainty (e.g. confidence intervals)
☐	☒	For null hypothesis testing, the test statistic (e.g. F, t, r) with confidence intervals, effect sizes, degrees of freedom and P value noted<br>*Give P values as exact values whenever suitable.*
☒	☐	For Bayesian analysis, information on the choice of priors and Markov chain Monte Carlo settings
☒	☐	For hierarchical and complex designs, identification of the appropriate level for tests and full reporting of outcomes
☒	☐	Estimates of effect sizes (e.g. Cohen's d, Pearson's r), indicating how they were calculated

Our web collection on statistics for biologists contains articles on many of the points above.

Software and code

Policy information about availability of computer code

Data collection	Flow cytometry data were acquired using the Canto and Diva software programs installed on our clinical FACS Canto-II machines.
Data analysis	Flow cytometry data were analyzed using Flow Jo (version ≥10.6) (BD Biosciences, San Jose, CA). A subset of the flow cytometry data were analyzed using CITRUS (Cluster Identification, Characterization, and Regression; Beckman Coulter, Brea, CA), an automated, unsupervised clustering algorithm.

For manuscripts utilizing custom algorithms or software that are central to the research but not yet described in published literature, software must be made available to editors and reviewers. We strongly encourage code deposition in a community repository (e.g. GitHub). See the Nature Research guidelines for submitting code & software for further information.

Data

Policy information about availability of data

All manuscripts must include a data availability statement. This statement should provide the following information, where applicable:

- Accession codes, unique identifiers, or web links for publicly available datasets
- A list of figures that have associated raw data
- A description of any restrictions on data availability

All relevant data are within the paper and its supporting (supplemental) information files.

Field-specific reporting

Please select the one below that is the best fit for your research. If you are not sure, read the appropriate sections before making your selection.

☒ Life sciences ☐ Behavioural & social sciences ☐ Ecological, evolutionary & environmental sciences

For a reference copy of the document with all sections, see nature.com/documents/nr-reporting-summary-flat.pdf

Life sciences study design

All studies must disclose on these points even when the disclosure is negative.

Sample size	No sample size calculation was performed. The number of HIV+ study subjects (n=6 in each of the two HIV+ study subject cohorts) was selected based on the number of patients in our HIV clinic that met the study criteria and consented to the study. The number of HIV negative subjects (n=11) recruited into the study was based on matching the total number of HIV+ study subjects (n=12).
Data exclusions	No data were excluded.
Replication	Due to IRB mandated sample collection limits from pediatric subjects, each study subject sample was evaluated once for the study.
Randomization	The HIV+ study subjects were divided into two groups, Cohorts 1 and 2, based on the longitudinal pattern of virological control (see Figure 1). Cohort 1 displayed better virological control versus Cohort 2.
Blinding	Flow cytometry samples were blinded to the group classification (Cohort 1 or Cohort 2) of the HIV+ study subjects at the time of sample acquisition. Serum samples for the childhood vaccine-specific antibody studies were aliquoted and stored using a coding system. The laboratory staff performing the data collection on the serum aliquots were blinded to the grouping information of the study subjects.

Reporting for specific materials, systems and methods

We require information from authors about some types of materials, experimental systems and methods used in many studies. Here, indicate whether each material, system or method listed is relevant to your study. If you are not sure if a list item applies to your research, read the appropriate section before selecting a response.

Materials & experimental systems

n/a	Involved in the study	
☐	☒	Antibodies
☒	☐	Eukaryotic cell lines
☒	☐	Palaeontology and archaeology
☐	☐	Animals and other organisms
☐	☒	Human research participants
☐	☒	Clinical data
☒	☐	Dual use research of concern

Methods

n/a	Involved in the study	
☒	☐	ChIP-seq
☐	☒	Flow cytometry
☒	☐	MRI-based neuroimaging

Antibodies

Antibodies used

(a) BD Multitest™ 6-color TBNK reagent:CD3 FITC / CD16 PE + CD56 PE / CD45 PerCP-Cy™5.5 / CD4 PE-Cy™7 / CD19 APC / CD8 APC-Cy™7. Catalog Number: 662967 (BD Biosciences). This is an IVD product. Multiple lot numbers have been used for clinical testing of the patient specimens over the years. Current Lot Number is: 30568
(b) BD Pharmingen™ Pacific Blue™ Mouse Anti-Human CD3; Clone UCHT1; Lot No: 9332900; Catalog No.: 558117.
(c) Biolegend PE/Cyanine7 anti-human IFN-γ Antibody; Clone 4S.B3; Lot Number:B256481. Catalog No.: 502528.
(d) Biolegend PE/Cyanine7 Mouse IgG1, κ Isotype Ctrl Antibody; Clone MOPC-21; Lot Number:B243399. Catalog No.: 400126
(e) Biolegend PE anti-human TNF-α Antibody; Clone MAb11; Lot Number:B250947. Catalog No.: 502909.
(f) Biolegend PE Mouse IgG1, κ Isotype Ctrl (ICFC) Antibody; Clone MOPC-21; Lot Number:B244869. Catalog No.: 400140.
(g) Biolegend PerCP/Cyanine5.5 anti-human IL-2 Antibody; Clone MQ1-17H12; Lot Number:B290822 Catalog No.: 500322
(h) Biolegend PerCP/Cyanine5.5 Rat IgG2a, κ Isotype Ctrl Antibody; Clone RTK2758; Lot Number:B268003; Catalog No.: 400531
(i) Biolegend Alexa Fluor® 647 anti-human IL-21 Antibody; Clone 3A3-N2; Lot Number:B260495; Catalog No.: 513006
(k) Biolegend Alexa Fluor® 647 Mouse IgG1, κ Isotype Ctrl (FC) Antibody; Clone MOPC-21; Lot Number:B277417; Catalog No.: 400130
(l) Biolegend PE anti-human CD8a Antibody; Clone RPA-T8; Lot Number:B262057; Catalog No.: 301008.
(m) BD Pharmingen™ APC-H7 Mouse anti-Human CD8; Clone SK1; Lot Number:9074798; Catalog No.: 560179
(n) BD Phosflow™ Alexa Fluor® 647 Mouse Anti-Stat5 (pY694); Clone: 47/Stat5(pY694); Lot Number:9192735; Catalog No.: 612599
(o) BD Phosflow™ Alexa Fluor® 647 Mouse Anti-Stat1 (pY701); Clone:4a; Lot Number:8094785; Catalog No.: 612597
(p) BD Phosflow™ Alexa Fluor® 647 Mouse Anti-Stat6 (pY641); Clone:18/P-Stat6; Lot Number:7347671; Catalog No.: 612601
(q) BD Phosflow™ Alexa Fluor® 647 Mouse Anti-Stat3 (pY705); Clone:4/P-STAT3; Lot Number:7346960; Catalog No.: 557815
(r) BD Phosflow™ Alexa Fluor® 647 Mouse Anti-ZAP70 (PY319)/Syk (PY352); Clone:17A/P-ZAP70; Lot Number:7338531; Catalog No.: 557817

(s) BD Pharmingen™ PE Mouse Anti-Human CD107a; Clone:H4A3; Lot Number:8130821; Catalog No.: 555801
(t) BD™ Mouse IgG1 PE; Clone:X40; Lot Number:9050727; Catalog No.: 340761
(u) Invitrogen CD28 Monoclonal Antibody (Clone: CD28.2), Functional Grade, eBioscience™; Lot Number:2120160; Catalog No.: 16-0289-85
(v) Invitrogen CD49d (Integrin alpha 4) Monoclonal Antibody (Clone: 9F10), Functional Grade, eBioscience™; Lot Number:2062915; Catalog No.: 16-0499-85
(w)Biolegend LEAF™ Purified Mouse IgG1, κ Isotype Ctrl Antibody; Clone: MOPC-21; Lot Number:B236633; Catalog No.: 400124
(x) Biolegend LEAF™ Purified anti-human CD3 Antibody; Clone: UCHT1; Lot Number:B233510; Catalog No.: 300414.
(y) Biolegend Purified Goat anti-mouse IgG (minimal x-reactivity) Antibody; Clone: Poly4053; Lot Number:B205395; Catalog No.: 405301.
(z) Biolegend PerCP/Cyanine5.5 anti-human CD3 Antibody; Clone: SK7; Lot Number:B253484; Catalog No.: 344807
(aa) Biolegend PE anti-human CD19 Antibody; Clone: HIB19; Lot Number:B273506; Catalog No.: 302208
(bb) Biolegend Alexa Fluor® 647 anti-human CD4 Antibody; Clone: SK3; Lot Number:B276518; Catalog No.: 344636.
(cc) BD Phosflow™ Alexa Fluor® 647 Mouse Anti-Stat4 (pY693); Clone:38/p-Stat4; Lot Number:8128626; Catalog No.: 558137.
.

.

Validation

All antibodies were validated by manufacturer.

Animals and other organisms

Policy information about studies involving animals; ARRIVE guidelines recommended for reporting animal research

Laboratory animals

Not applicable.

Wild animals

Not applicable.

Field-collected samples

Not applicable.

Ethics oversight

Not applicable.

Note that full information on the approval of the study protocol must also be provided in the manuscript.

Human research participants

Policy information about studies involving human research participants

Population characteristics

Please refer to supplementary table 1 for study subject related demographics and data.

Recruitment

HIV+ pediatric study subjects recruited into the study were selected on the basis of the differences in longitudinal viral load measurements, and timing of initiation of anti-retroviral therapy. The HIV negative study subjects were recruited to establish symmetry in the age-range and gender distribution of the HIV+ study subjects.

Ethics oversight

The Institutional Review Board (IRB) at the Ann and Robert H. Lurie Children's Hospital of Chicago approved the study.

Note that full information on the approval of the study protocol must also be provided in the manuscript.

Clinical data

Policy information about clinical studies
All manuscripts should comply with the ICMJE guidelines for publication of clinical research and a completed CONSORT checklist must be included with all submissions.

Clinical trial registration

Not applicable

Study protocol

Not applicable

Data collection

The data were collected at the Ann and Robert H. Lurie Children's Hospital of Chicago between July 2018 and March of 2020.

Outcomes

(a) Defined functional properties of the circulating lymphocytes in the study subjects, including intracellular cytokine responses, markers of degranulation, phosphoprotein signatures in response to stimulation with immunomodulatory cytokines. (b) We also measured antibody responses to four childhood vaccines.

Flow Cytometry

Plots

Confirm that:

☒ The axis labels state the marker and fluorochrome used (e.g. CD4-FITC).

☒ The axis scales are clearly visible. Include numbers along axes only for bottom left plot of group (a 'group' is an analysis of identical markers).

☒ All plots are contour plots with outliers or pseudocolor plots.

☒ A numerical value for number of cells or percentage (with statistics) is provided.

Methodology

Sample preparation	For functional studies, whole blood samples collected in Na-heparin treated blood collection tubes were treated with the described stimulating agents within 4-6 hours of blood draw. Antibody responses to childhood vaccines were evaluated in thawed serum samples obtained from the study subjects. Serum samples were harvested from whole blood collected in blood collection tubes devoid of any anticoagulants, within 2 hours of blood draw and the aliquots were frozen at -80C.
Instrument	Flow cytometry data were acquired using FACS-Canto II flow cytometers (BD Biosciences).
Software	Data were analyzed using Flow Jo software (version 10.6 and higher). Phosflow data were analyzed using Cytobank software.
Cell population abundance	We did not sort any populations for this study.
Gating strategy	The gating strategy is depicted in Supplementary Figure 1. The demarcations between positive and negative populations for the degranulation marker, CD107a, as well as for the phosphoproteins are clearly defined in the relevant figures.

☒ Tick this box to confirm that a figure exemplifying the gating strategy is provided in the Supplementary Information.
