# Supplementary material for: Preservation of lymphocyte functional fitness in perinatally-infected and treated HIV+ pediatric patients displaying sub-optimal viral control
Source: Commun Med (Lond). Author manuscript; Available in PMC 2022 Apr 15. (PMC9012494; doi:10.1038/s43856-022-00085-9)
Supplement: Supplementary file 2 [file NIHMS1787374-supplement-Supplementary_Information.pdf]

Supplementary Table 1. Study subject information

	HIV+		HIV-
	Cohort-1	Cohort-2	
Gender			
Male	4	4	6
Female	2	2	5
Age at enrollment			
0-5 years	2	1	3
6 – 10 years	2	1	4
≥ 11 years	2	4	4
Age at HIV diagnosis			
Not known[1]	0	2	N/A[2]
≤ 6 weeks	2	1	
6 weeks – 6 months	3	1	
6 – 12 months	0	2	
12 – 15 months	1	0	
Age at ART initiation			
≤ 6 weeks	2	0	N/A
6 weeks – 6 months	3	3	
6 – 12 months	0	3	
12 – 15 months	1	0	
Median age at the time of ART initiation, weeks (range)	10.4 (0.1-58.7)	32.50 (6.4-46.6)	
Median age at time of initial viral suppression[3], weeks (range)	23* (1-84)	57* (25-99)	N/A
Number of participants with genotypic resistance to ARTs[4]			
Never tested	2	0	N/A
Wild-type virus detected	4	0	
Resistance to NRTI	0	6	
Resistance to NNRTI	0	5	
Resistance to PI	0	3	
Median age at the time of blood draw for this study, years (range)	10.3 (5-17)	15.9 (4-24)	7 (2-18)

[1]Patients transferred care from other provider, records do not indicate age diagnosis established
[2]N/A: Not applicable
[3]Viral suppression defined as time when viral load first measured ≤200 copies/mL
[4]Number of participants ever documented with resistance mutations for at least one drug in class; NRTI=nucleoside/nucleotide reverse transcriptase inhibitors, NNRTI=non-nucleoside reverse transcriptase inhibitors, PI=protease inhibitor
*p-value: 0.0411

Supplementary Table 2. Background frequency of cytokine production observed in the intracellular cytokine staining assays depicted in Figures 2 and 5.

	Cohort-1 (n=6)*	Cohort-2 (n=6)*	Healthy Controls (n=11)*
Tube A CD8+ T cells†	0.008530± 0.02468%	0.01271± 0.08651%	0.004341± 0.01121%
Tube B CD8+ T cells†	0.01783± 0.04137%	0.04456± 0.1513%	0.01231± 0.03567%
Tube C CD8+ T cells†	0.005880± 0.02047%	0.007394± 0.03410%	NA
Tube A CD8- T cells†	0.01104± 0.02766%	0.02313± 0.1762%	0.006433± 0.02320%
Tube B CD8- T cells†	0.01634± 0.03800%	0.04731± 0.2087%	0.009662± 0.02922%
Tube A CD3 negative lymphocytes†	0.01470± 0.04207%	0.03775± 0.2864%	0.006843± 0.01361%
Tube B CD3 negative lymphocytes†	0.02382± 0.06584%	0.08397± 0.4717%	0.01521± 0.04777%

Tube A: unstimulated samples stained with the antibodies for IFNγ, TNFα, IL-2, IL-21; Tube B: PMA/Ion stimulated samples and stained with the relevant, dose-matched isotype control Ab for IFNγ, TNFα, IL-2, IL-21; Tube C: HIV Gag PTE pool stimulated samples and stained with the relevant, dose-matched isotype control Ab for IFNγ, TNFα, IL-2, IL-21. *Numeric values represent the mean ± SD of 90 separate cytokine combinations each for cohorts 1 and 2 (15 combinations per donor), and 165 separate cytokine combinations for the healthy control group (15 combinations per donor). NA; Not applicable. †No statistically significant differences noted among or between the groups.

Supplementary Table 3. ART regimens at enrollment

Regimen[1]					Cohort-1	Cohort-2
NRTI	NNRTI	PI	INSTI	Booster		
Emtricitabine Tenofovir			Elvitegravir	Cobicistat	2	1
Emtricitabine Tenofovir	Rilpivirine				1	0
Abacavir Lamivudine		Lopinavir/ ritonavir			1	0
Zidovudine Lamivudine	Nevirapine				1	
Abacavir Emtricitabine			Raltegravir		1	0
Abacavir Lamivudine			Dolutegravir		0	1
Zidovudine Abacavir			Raltegravir		0	2
	Rilpivirine	Darunavir	Dolutegravir	Cobicistat	0	1
Emtricitabine Tenofovir	Rilpivirine		Dolutegravir		0	1

[1]NRTI: nucleoside/nucleotide reverse transcriptase inhibitor; NNRTI: non-nucleoside reverse transcriptase inhibitor; PI: protease inhibitor; INSTI: integrase strand transfer inhibitor

Supplementary Figure 1

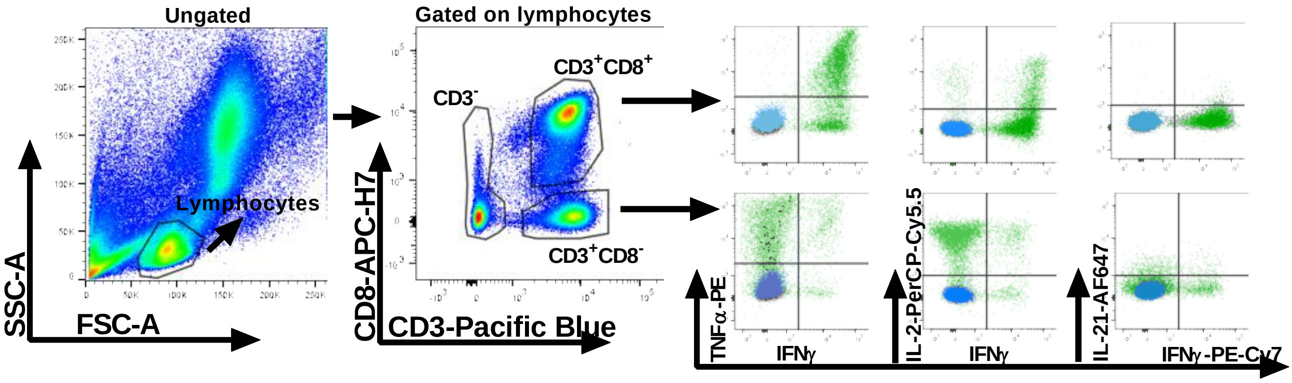

Boolean Gating: 2^{4} combinations

Intracellular Cytokine Combinations	CD3^{+}CD8^{+} T cells	CD3^{+}CD8^{-} T cells	CD3 negative
IFNγ^{+}IL-2^{+}IL-21^{+}TNFα^{+}	X	X	X
IFNγ^{+}IL-2^{+}IL-21^{+}TNFα^{-}	X	X	X
IFNγ^{+}IL-2^{+}IL-21^{-}TNFα^{+}	X	X	X
IFNγ^{+}IL-2^{+}IL-21^{-}TNFα^{-}	X	X	X
IFNγ^{+}IL-2^{-}IL-21^{+}TNFα^{+}	X	X	X
IFNγ^{+}IL-2^{-}IL-21^{+}TNFα^{-}	X	X	X
IFNγ^{+}IL-2^{-}IL-21^{-}TNFα^{+}	X	X	X
IFNγ^{+}IL-2^{-}IL-21^{-}TNFα^{-}	X	X	X
IFNγ^{-}IL-2^{+}IL-21^{+}TNFα^{+}	X	X	X
IFNγ^{-}IL-2^{+}IL-21^{+}TNFα^{-}	X	X	X
IFNγ^{-}IL-2^{+}IL-21^{-}TNFα^{+}	X	X	X
IFNγ^{-}IL-2^{+}IL-21^{-}TNFα^{-}	X	X	X
IFNγ^{-}IL-2^{-}IL-21^{+}TNFα^{+}	X	X	X
IFNγ^{-}IL-2^{-}IL-21^{+}TNFα^{-}	X	X	X
IFNγ^{-}IL-2^{-}IL-21^{-}TNFα^{+}	X	X	X
IFNγ^{-}IL-2^{-}IL-21^{-}TNFα^{-}	X	X	X

Supplementary Figure 2

a.

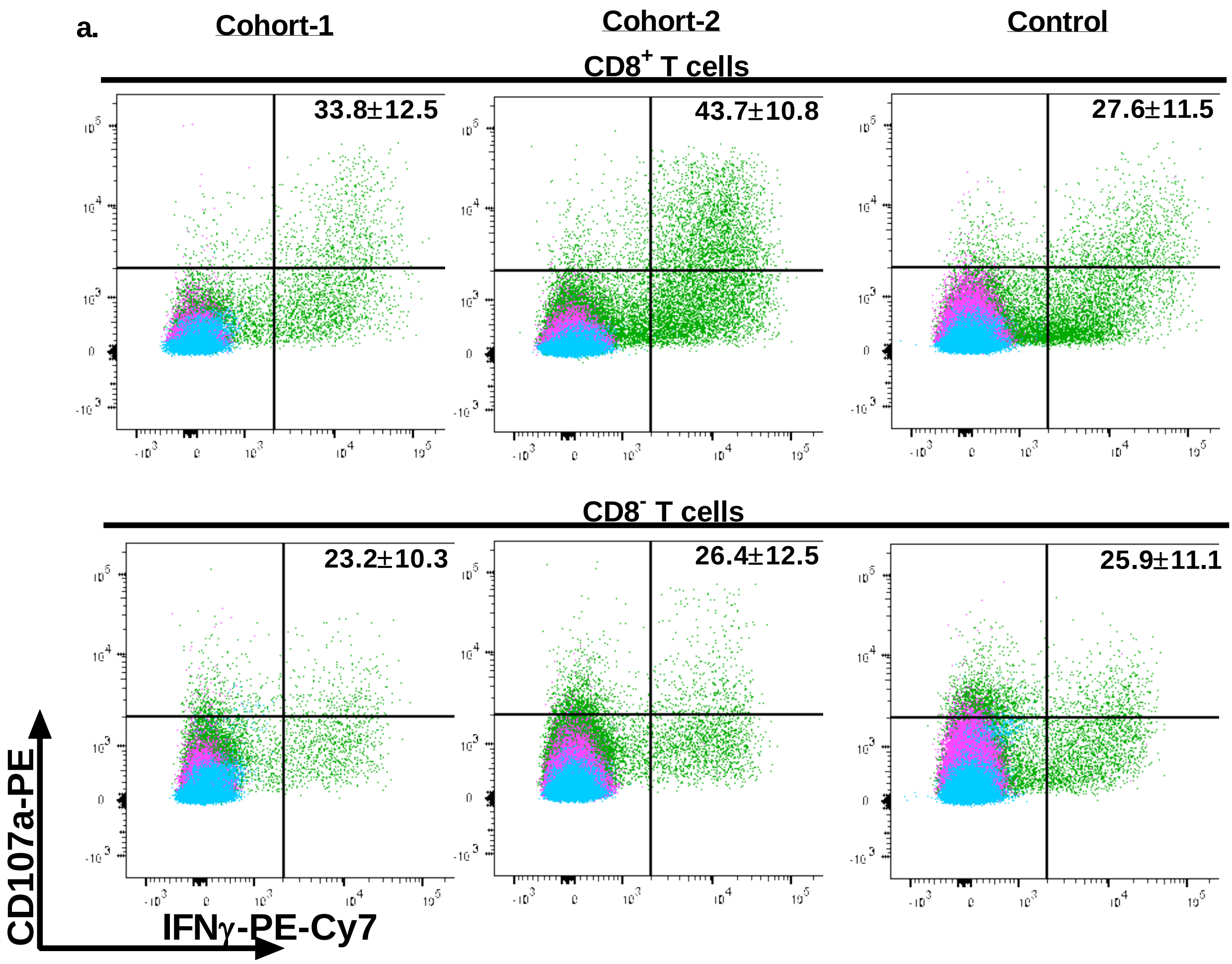

b.

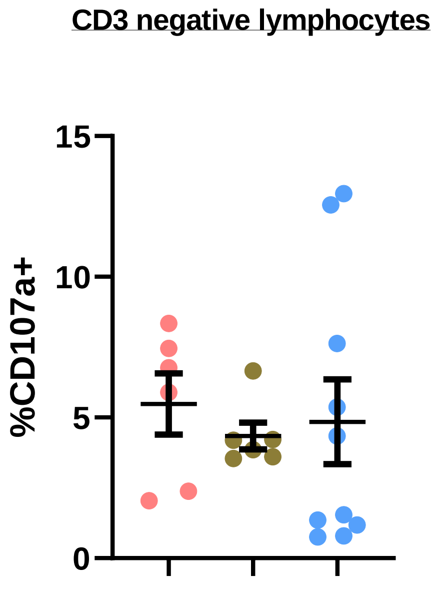

Supplementary Figure 3

a.

CITRUS Analysis (Medians)

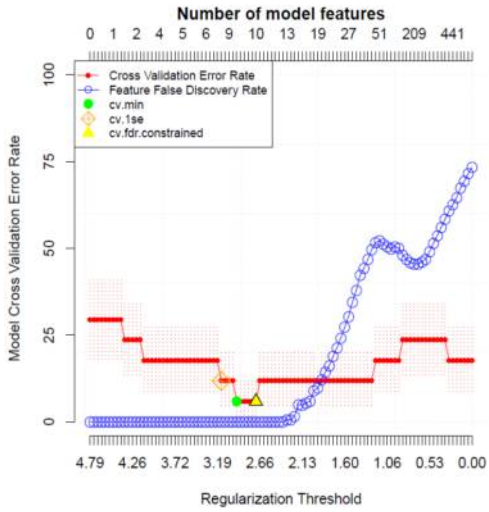

Feature Plots cv.1se

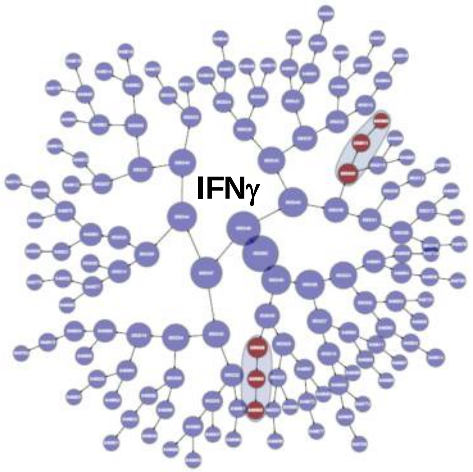

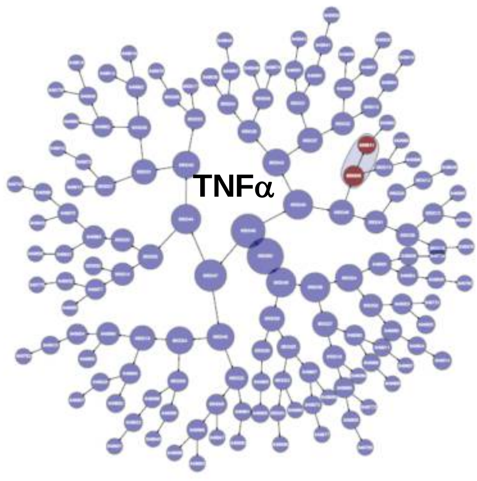

b.

CITRUS Analysis (Abundance)

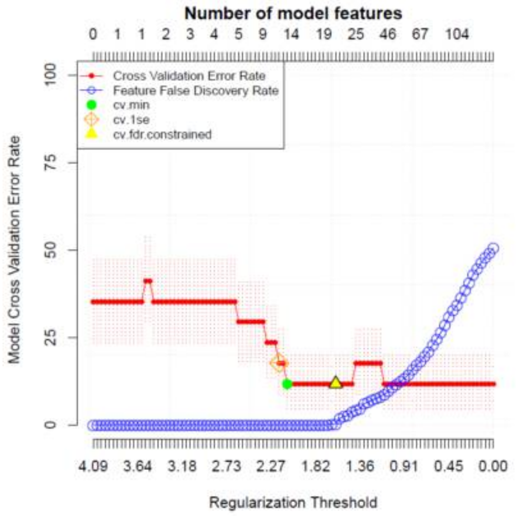

Feature Plots cv.1se

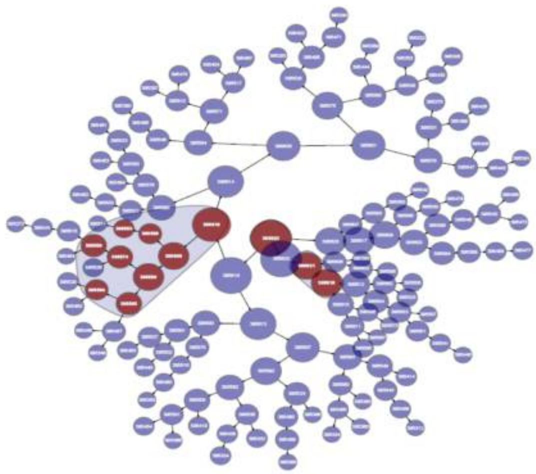

Supplementary Figure 4

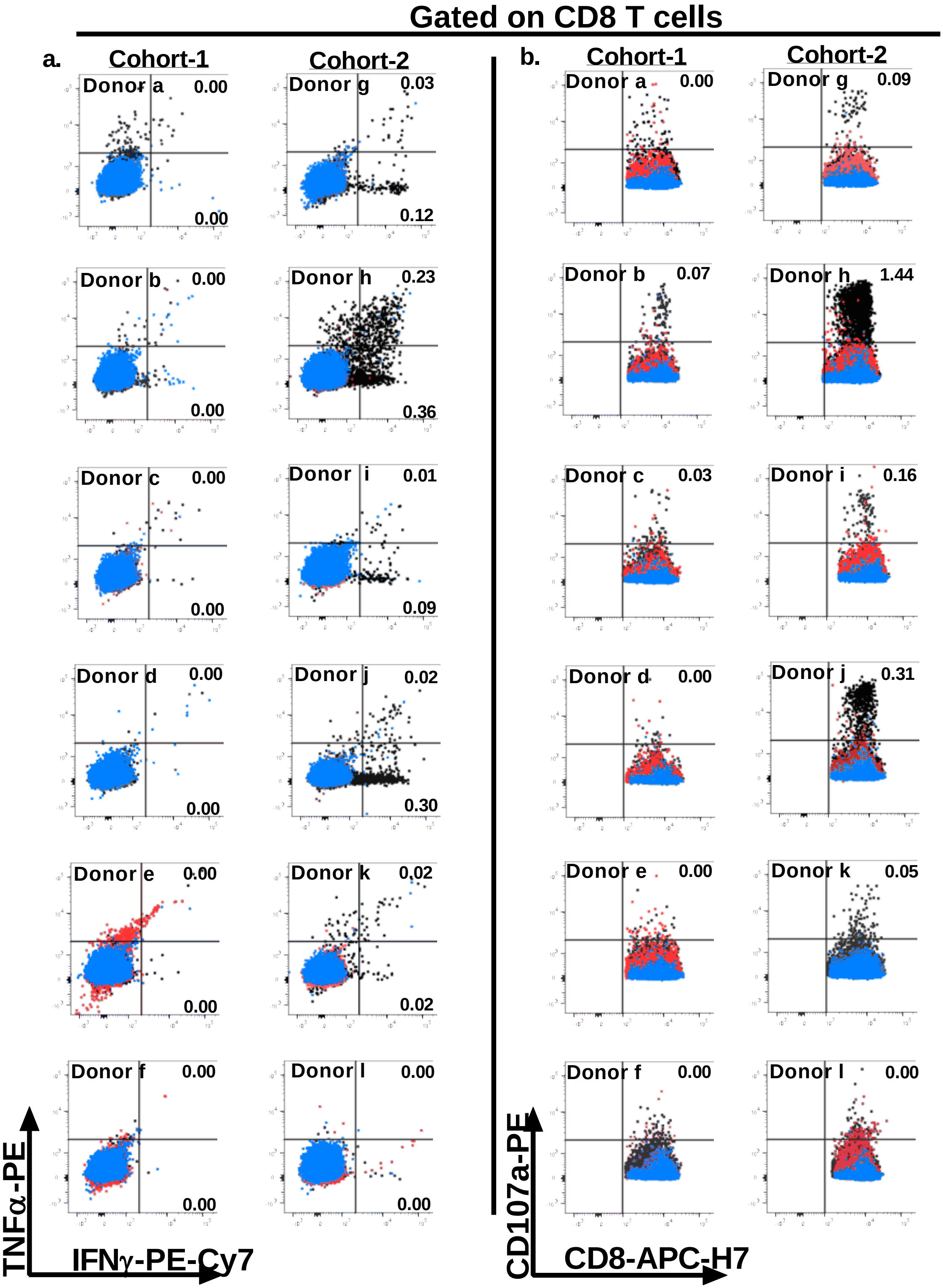

Supplementary Figure 5

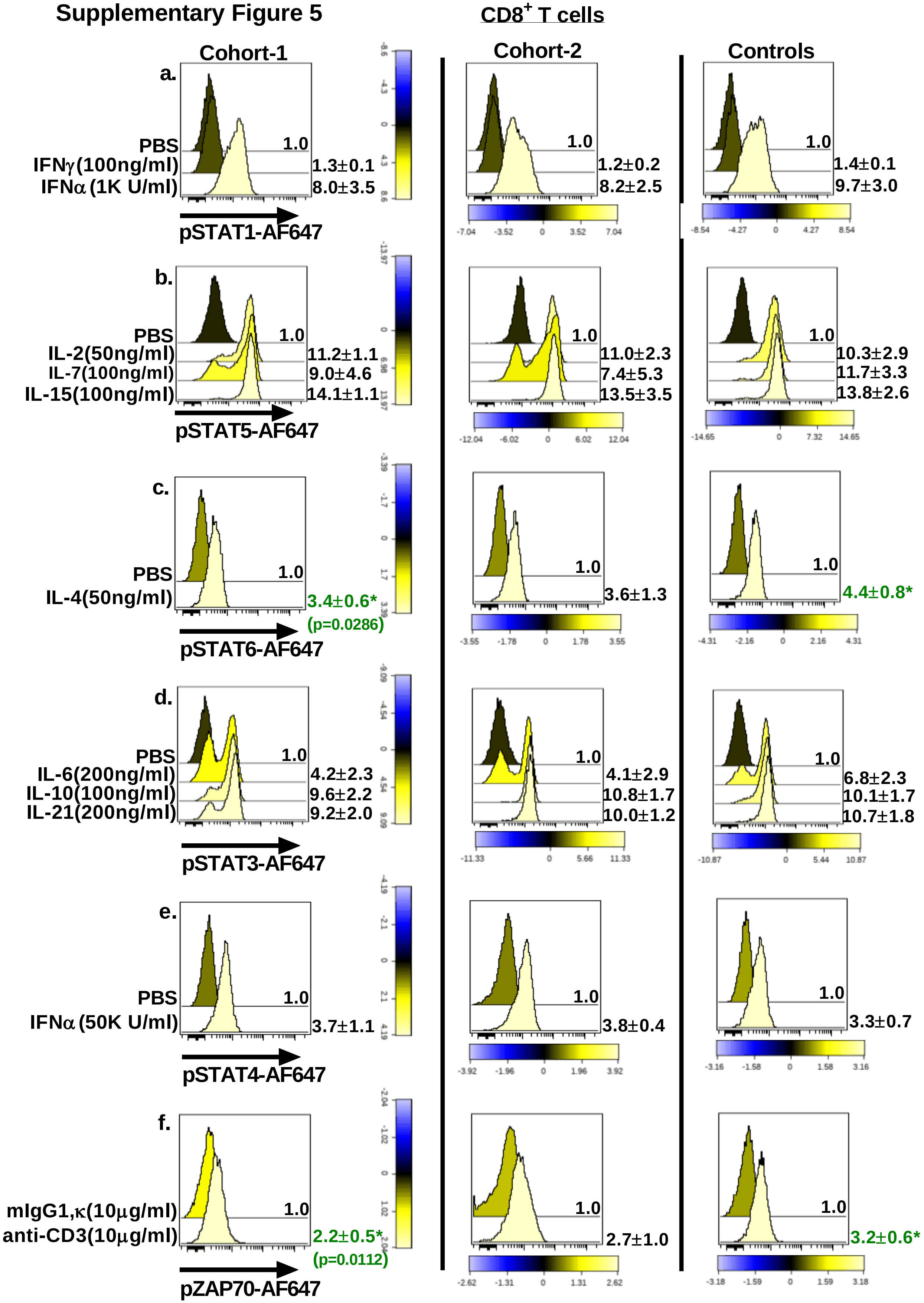

Supplementary Figure 6

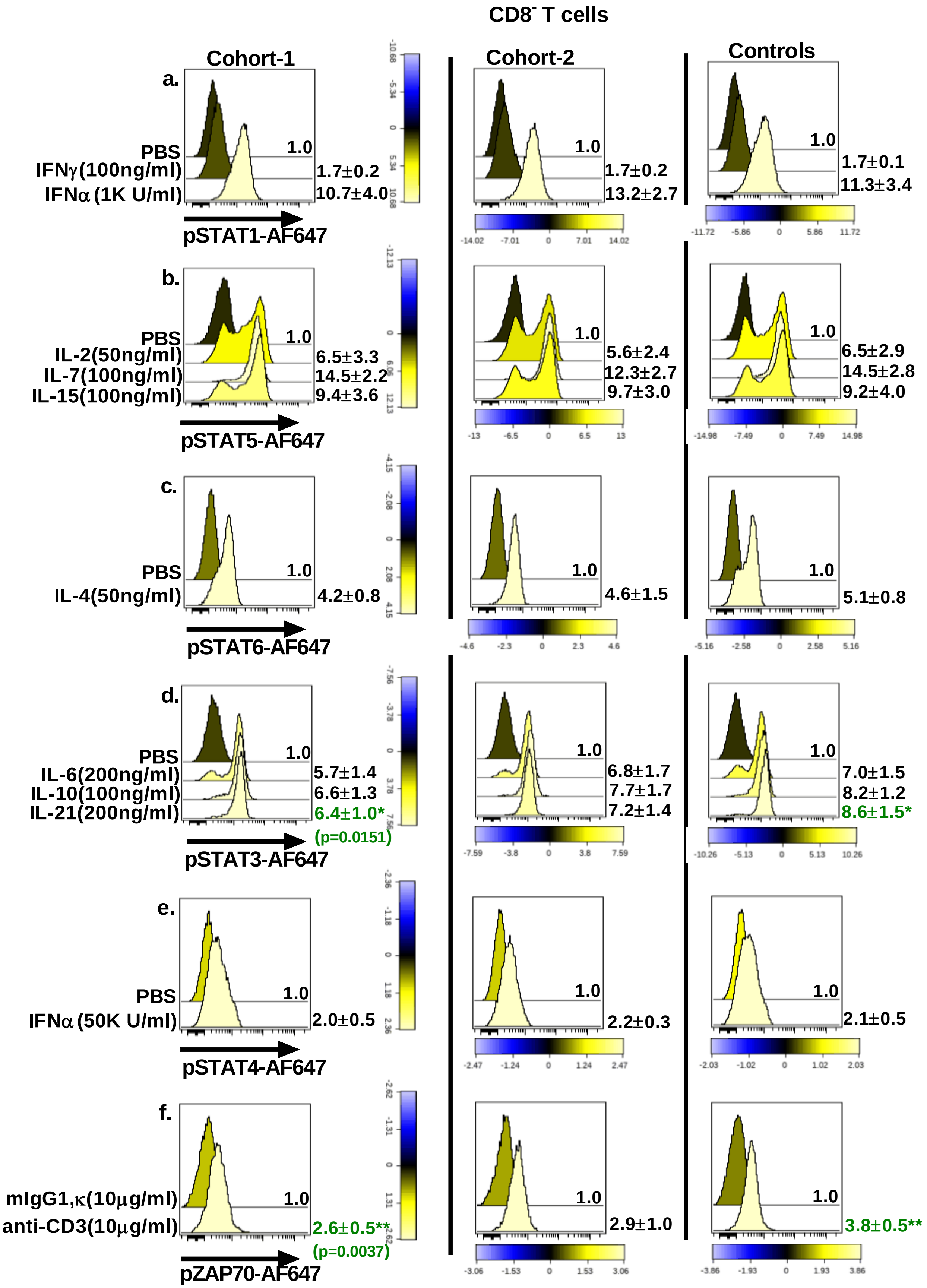

Supplementary Figure 7

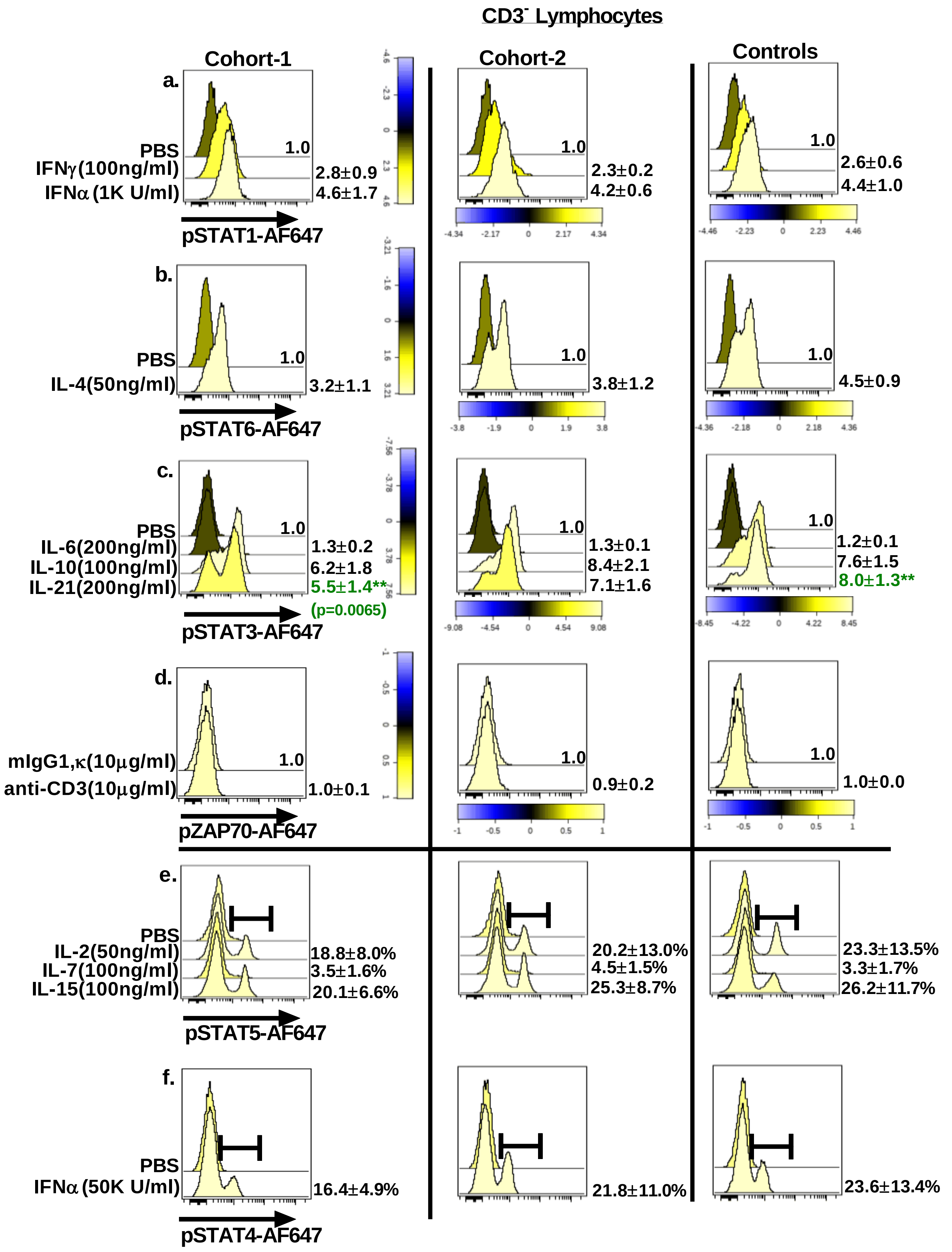

Supplementary Figure 8

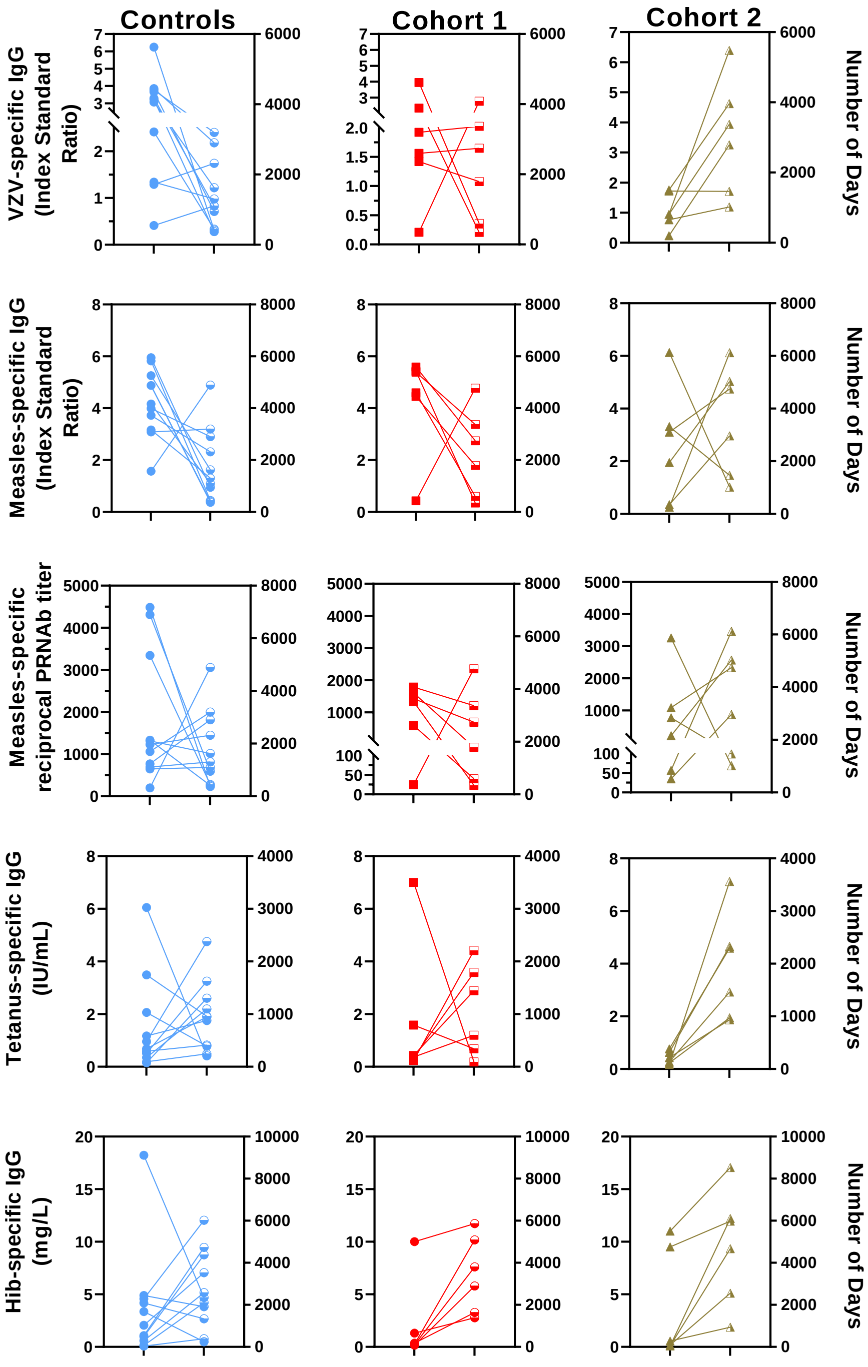

Supplementary Figure 1. Gating strategy for the mitogen-stimulated intracellular cytokine secretion assay. Lymphocytes were identified based on light-scatter characteristics, and hierarchical gating of T cell subsets and CD3 negative lymphocytes was performed with the aid of lineage-directed MAbs. Polyfunctionality was ascertained by using a cocktail of four MAbs targeting cytosolic IFNγ, TNFα, IL-2 and IL-21. Boolean gating was employed to identify the measurable combinations of the four cytokines produced by the gated lymphocyte subsets. Green dots: cells that are PMA/Ion stimulated and stained with MAbs against IFNγ, TNFα, IL-2 and IL-21. Blue dots: cells that are PMA/Ion stimulated and stained with isotype-control Abs against IFNγ, TNFα, IL-2 and IL-21. Pink dots: cells that are unstimulated and stained with MAbs against IFNγ, TNFα, IL-2 and IL-21.

Supplementary Figure 2. Examination of the degranulation potential of mitogen-stimulated lymphocyte subsets. (a) Surface mobilization of CD107a was measured in tandem with intracellular IFNγ production with and without PMA/Ion stimulation for the indicated T cell subsets. The numerical values in the upper-right quadrants of the dot-plots depict the background-subtracted frequencies (mean % ± SD) of the gated T cells co-expressing CD107a and IFNγ as a proportion of the total IFNγ producing cells. Green dots: cells that are PMA/Ion stimulated and stained with MAbs against CD107a and IFNγ. Blue dots: cells that are PMA/Ion stimulated and stained with isotype-control Abs against CD107a and IFNγ. Pink dots: cells that are unstimulated and stained with MAbs against CD107a and IFNγ. (b) The scatter plot graphs represent the background-subtracted frequencies of CD3 negative lymphocytes mobilizing CD107a to the cell-surface following PMA/Ion stimulation. Data for individual subjects are displayed along with mean±SEM for each group. Pink circles: Cohort 1; Green circles: Cohort 2; Blue circles: Control group. Each study subject was evaluated once. Cohort 1 (n=6 biologically independent samples), Cohort 2 (n=6 biologically independent samples), Controls (n=11 biologically independent samples).

Supplementary Figure 3. CITRUS analyses of PMA/Ion stimulated CD3 positive and negative lymphocytes based on the median (a) and abundance (b) criterion. The minimum cluster size setting used was 1% with 10 cross-validation folds (CVF), and a false discovery rate (FDR) of 1% for both the median and abundance features. Top to bottom in each column: the model error rate followed by the cluster diagrams where the maroon clusters represent subsets of lymphocytes that are significantly different between the healthy controls and cohort 1 HIV+ study subjects. These profiles are representative of four independent CITRUS runs (comparing the HIV negative control subjects versus cohort 1 subjects) separately examining the median expression of the cytokines, and the abundance of the clusters that are significantly enriched in cohort 1. Cohort 1 (n=6 biologically independent samples), Controls (n=11 biologically independent samples).

Supplementary Figure 4. Visualization of HIV-specific CD8 T cell responses. First two columns: Intracellular cytokine staining was performed with and without stimulation of whole blood samples with the HIV-Gag PTE peptide pool. The numerical values in each plot denote background-subtracted frequencies of CD8 T cells producing IFNγ alone (lower-right quadrant) and co-producing IFNγ and TNFα (upper-right quadrant). Third and fourth columns: The degranulation potential of Gag-specific CD8 T cells was evaluated by measuring the surface mobilization of CD107a with and without stimulation of whole blood samples with HIV-Gag PTE peptide pool. The numerical values in the upper-right quadrants depict the background-subtracted frequencies of Gag-specific $CD8^{+}CD107a^{+}$ T cells. Black dots: cells that are Gag-PTE pool stimulated and stained with MAbs against CD107a, IFNγ and TNFα. Blue dots: cells that are Gag-PTE pool stimulated and stained with isotype-control Abs against CD107a, IFNγ and TNFα. Red dots: cells that are unstimulated and stained with MAbs against CD107a, IFNγ and TNFα. Each study subject was evaluated once. Cohort 1 (n=6 biologically independent samples), Cohort 2 (n=6 biologically independent samples).

Supplementary Figure 5. Phosphoprotein signature of bulk CD8(+) T cells. Whole blood samples were treated with PBS or the indicated cytokines or anti-CD3 MAb. The numerical values in each stacked histogram plot denote the mean±SD of the fold change in the MFI of the phosphorylation signal of the signaling node following treatment with the signaling input compared to PBS treatment which is normalized to one. The change in the phosphorylation status is additionally depicted by the color of each histogram based on the colorimetric scale placed below and/or adjacent to each plot. p-values < 0.05 are considered statistically significant. Each study subject was evaluated once. Cohort 1 (n=6 biologically independent samples), Cohort 2 (n=6 biologically independent samples), Controls (n=11 biologically independent samples). AF647: Alexa Fluor 647.

Supplementary Figure 6. Phosphoprotein signature of bulk CD8(-) T cells. Whole blood samples were treated with PBS or the indicated cytokines or anti-CD3 MAb. The numerical values in each stacked histogram plot denote the mean±SD of the fold change MFI of the phosphorylation signal of the signaling node following treatment with the signaling input compared to PBS treatment which is normalized to one. The change in the phosphorylation status is additionally depicted by the color of each histogram based on the colorimetric scale placed below and/or adjacent to each plot. p-values < 0.05 are considered statistically significant. Each study subject was evaluated once. Cohort 1 (n=6 biologically independent samples), Cohort 2 (n=6 biologically independent samples), Controls (n=11 biologically independent samples). AF647: Alexa Fluor 647.

Supplementary Figure 7. Phosphoprotein signature of bulk CD3(-) lymphocytes. Whole blood samples were treated with PBS or the indicated cytokines or anti-CD3 Ab. The numerical values in each stacked histogram plot denote the mean±SD of the fold change MFI of the phosphorylation signal of the signaling node following treatment with the signaling input compared to PBS treatment which is normalized to one. The change in the phosphorylation status is additionally depicted by the color of each histogram based on the colorimetric scale placed below and/or adjacent to each plot. Note: For IL-

2, IL-7, IL-15 and IFNα-50K U/ml, the data are depicted as the means±SD of the background-subtracted frequencies rather than as fold-change in MFI because a weak bimodal signal was observed following cytokine treatment. Hence displaying the data as % rather than as fold change in MFI was more informative in these instances. p-values < 0.05 are considered statistically significant. Each study subject was evaluated once. Cohort 1 (n=6 biologically independent samples), Cohort 2 (n=6 biologically independent samples), Controls (n=11 biologically independent samples). AF647: Alexa Fluor 647.

Supplementary Figure 8. Relationship between serum Ab levels and time elapsed since latest vaccine dose. Serum Ab levels for each study subject for all four vaccines are indicated by filled symbols and are listed along the left y-axes. The time intervals between the latest vaccine dose and the serum Ab assessment for each study subject for all four vaccines are indicated by half-filled symbols and are listed along the right y-axes. Blue circles: Control subjects; Red squares: Cohort 1 subjects; Green triangles: Cohort 2 subjects. Each study subject was evaluated once. Cohort 1 (n=6 biologically independent samples), Cohort 2 (n=6 biologically independent samples), Controls (n=11 biologically independent samples).
